# NDR/LATS‐family protein kinase genes are indispensable for embryogenesis in Arabidopsis

**DOI:** 10.1002/2211-5463.13257

**Published:** 2021-08-06

**Authors:** Hyuk Sung Yoon, Kaien Fujino, Shenkui Liu, Tetsuo Takano, Daisuke Tsugama

**Affiliations:** ^1^ Asian Research Center for Bioresource and Environmental Sciences Graduate School of Agricultural and Life Sciences The University of Tokyo Nishitokyo‐shi Japan; ^2^ Laboratory of Crop Physiology Research Faculty of Agriculture Hokkaido University Sapporo‐shi Japan; ^3^ State Key Laboratory of Subtropical Silviculture Zhejiang A & F University Lin’an China

**Keywords:** 14‐3‐3 protein, *Arabidopsis thaliana*, embryogenic lethality, phosphorylation, protein kinase

## Abstract

NDR/LATS‐family protein kinases are conserved among eukaryotes. These protein kinases in yeast and animals phosphorylate specific targets and regulate the cell cycle. *Arabidopsis thaliana* has eight NDR/LATS‐family protein kinase genes (*NDR1‐8*), of which *NDR2*, *NDR4*, and *NDR5* are involved in regulating pollen development. However, the functions of the other NDR/LATS‐family protein kinase genes in plants are unclear. Here, we show that three putative phosphorylation sites of an Arabidopsis basic leucine zipper transcription factor, VIP1, correspond to NDR/LATS‐family protein kinase phosphorylation motifs and that two of these three sites are phosphorylated by NDR2, NDR3, or NDR8 *in vitro*. Expression of *NDR1‐8* was detected in various tissues. An *NDR4 NDR6 NDR7 NDR8* quadruple mutation caused embryonic lethality These results suggest that different NDR/LATS‐family protein kinases in plants have distinct physiological roles.

## Graphical abstract

*Arabidopsis thaliana* has eight NDR/LATS‐family protein kinase genes, *NDR1‐8*. *NDR2*, *NDR4*, and *NDR5* regulate pollen development, but the functions of the others are unknown. A genetic analysis conducted in this study showed that *NDR4*, *NDR6*, *NDR7*, and *NDR8* regulate embryogenesis. This study also shows that an Arabidopsis transcription factor, VIP1, can be a substrate of at least NDR2, NDR3, and NDR8.

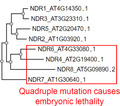

**Abbreviations**
AGC: protein kinases A, G, and C
GST: glutathione S‐transferase
LATS: large tumor suppressor
MBP: maltose‐binding protein
NDR: nuclear Dbf2‐related
PK: protein kinase
RT‐PCR: reverse transcription‐PCR
T‐DNA: transfer DNA

NDR (nuclear Dbf2‐related)/LATS (large tumor suppressor)‐family serine/threonine protein kinases (PKs) are members of the AGC (protein kinase A, G, and C) superfamily and conserved among eukaryotes including yeast, animals, and plants. Such proteins are involved in regulating the cell cycle in the budding yeast *Saccharomyces* *cerevisiae* [1], the fruit fly *Drosophila melanogaster* [2, 3], and *Homo sapiens* [4, 5]. In *H. sapiens*, two closely related transcriptional coactivators, YAP (Yes‐associated protein) and TAZ (transcriptional coactivator with PDZ binding motif), are phosphorylated by an NDR/LATS‐family PK, LATS2 [6, 7]. ASPP1 (apoptosis‐stimulating protein of p53‐1), which regulates functions of YAP and TAZ, is also phosphorylated by LATS2 [8, 9]. In *S*. *cerevisiae*, a transcription factor, Ace2 (activator of CUP1 expression 2), is phosphorylated by an NDR/LATS‐family PK, Cbk1 (cell wall biosynthesis kinase 1) [10]. Analyses of phosphorylation sites of YAP, TAZ, ASPP1, and Ace2 suggest that the consensus sequence for the NDR/LATS‐family PKs phosphorylate sites is Hx[RK]xx[ST] (where x is any amino acid, [RK] is either R or K, and [ST] is either S or T). This sequence is similar to consensus sequences for other PK families in the AGC superfamily. However, the NDR/LATS family is the only one that requires H at the beginning of the consensus sequence [11]. Phosphorylation in those sites in YAP and TAZ promotes interactions of these proteins with 14‐3‐3 proteins and thereby promotes cytoplasmic retention of YAP and TAZ to inhibit their transcriptional coactivation functions [6, 7]. NDR/LATS‐family PKs in *H. sapiens*, *S*. *cerevisiae*, and *D. melanogaster* are bound by Msp1‐one binder (MOB) proteins and thereby activated [12, 13, 14]. In *Arabidopsis thaliana*, NDR/LATS‐family PKs are encoded by eight genes, *NDR1‐8* [15]. Of these, *NDR2*, *NDR4*, and *NDR5* are more strongly expressed in pollen grains than in leaves, roots, and other vegetative tissues and are indispensable for pollen fertility. NDR2, NDR4, and NDR5 can interact with two Arabidopsis MOB proteins, MOB1A and MOB1B, and are thought to be activated by them [16]. However, neither substrates nor physiological roles for other plant NDR/LATS‐family PKs are clear.

VIP1 is an Arabidopsis basic leucine zipper transcription factor that interacts with 14‐3‐3 proteins that is retained in the cytoplasm by 14‐3‐3 proteins [17]. Here, we show that at least NDR2, NDR3, and NDR8 phosphorylate *in vitro* the Hx[RK]xx[ST] sequences in the 14‐3‐3 protein‐interaction sites in VIP1 and that an *NDR4 NDR6 NDR7 NDR8* quadruple mutation causes embryonic lethality.

## Materials and methods

### *In vitro* phosphorylation assays

Coding sequences of NDR2, NDR3, NDR8, and WAG2 were amplified by RT‐PCR using KOD FX Neo (Toyobo, Osaka, Japan), the cDNA from the seedlings (see the ‘Reverse transcription‐PCR (RT‐PCR)’ subsection below). The resulting PCR products were cloned into the pMAL‐c5E vector (New England Biolabs, Ipswich, MA, USA). Primers and restriction sites used to clone these genes are listed in Table S1. The resulting constructs were transformed into the *Escherichia coli* strain BL21(DE3). Maltose‐binding protein (MBP)‐fused forms of NDR2, NDR3, NDR8, and WAG2 (MBP‐NDR2, MBP‐NDR3, MBP‐NDR8, and MBP‐WAG2, respectively) were expressed in the *E. coli* cells and purified as previously described for MBP‐CPK21 [18]. GST‐fused VIP1 variants were expressed in *E. coli* cells and purified as previously described [18, 19]. *In vitro* phosphorylation assays were performed with these MBP‐fused proteins and GST‐fused proteins as previously described [18]. In these assays, phosphorylated proteins were detected by western blotting with Phos‐tag biotin (Fujifilm Wako, Osaka, Japan), MBP‐fused proteins were detected with an anti‐MBP monoclonal antibody (New England Biolabs) and an anti‐IgG (H + L chain) (mouse) pAb‐HRP (Medical & Biological Laboratories Co., Ltd, Tokyo, Japan), and GST‐fused proteins were detected with an HRP‐conjugated anti‐GST antibody (Fujifilm Wako). To quantify the levels of phosphorylation of the GST‐fused VIP1 variants, Phos‐tag biotin‐derived signals and the anti‐GST antibody‐derived signals for those proteins on the same membrane were measured as gray values on imagej [20] and expressed as relative values using the value for GST‐VIP1 as the reference (i.e., as 1). The relative values for the Phos‐tag biotin‐derived signals were divided by the anti‐GST antibody‐derived signals for the same samples, and resulting values were used as the phosphorylation levels of those proteins. These values were obtained from three individual replicates. Means and standard deviations (SDs) from these three replicates are presented in the figure.

### Reverse transcription‐PCR (RT‐PCR)

Rosette leaves, roots, flowers (floral buds and open flowers), and flower stalks (with neither floral buds nor open flowers) of five‐week‐old wild‐type plants and 10‐day‐old wild‐type seedlings were frozen in liquid nitrogen and ground to a fine powder with a mortar and pestle. Total RNA was extracted from the resulting powder with the NucleoSpin RNA Plant Kit (Macherey‐Nagel, Düren, Germany). cDNA was synthesized from 1 μg of the total RNA using the oligo (dT) primer and PrimeScript Reverse Transcriptase (Takara Bio, Kusatsu, Japan), diluted 20 times with distilled water, and used as PCR templates. The quantitative PCR was run with these templates, GoTaq qPCR Master Mix (Promega, Fitchburg, WI, USA), the StepOne Real‐Time PCR System (Thermo Fisher Scientific, Waltham, MA, USA), and the primers listed in Table S2. Relative expression levels were calculated with the comparative cycle threshold method using *UBQ5* as the internal control gene. Experiments were performed with three biological replicates.

### Plant materials and plant growth conditions

*Arabidopsis thaliana* ecotype Col‐0 was used as the wild‐type control for all experiments. Seeds of the lines that have transfer DNA (T‐DNA) in *NDR4*, *NDR6*, *NDR7*, and *NDR8* were provided by the Arabidopsis Biological Resource Center (ABRC, https://abrc.osu.edu/). Stock numbers of them were SALK_035906C, SALK_130076C, SALK_037043C, and SALK_075540C, respectively. These lines were crossed to generate T‐DNA insertion lines listed in Table 1. T‐DNA in these lines was analyzed as described in the ‘Genomic PCR’ subsection below. Plants were grown for 14 days on an agar medium (0.5 × Murashige and Skoog salts, 1% (w/v) sucrose, 2 mm MES (2‐morpholinoethanesulfonic acid), pH 5.8, and 0.8% (w/v) agar) and further grown on Rockwool cubes as previously described [21] to observe their phenotypes and to collect their seeds.

**Table 1:** T‐DNA insertion lines used in this study.

Line name	Description
*ndr4* (SALK_035906C)	Line with T‐DNA insertion in the first exon corresponding to the 5’ untranslated region of *NDR4*
*ndr6* (SALK_130076C)	Line with T‐DNA insertion in the ninth intron of *NDR6*
*ndr7* (SALK_037043C)	Line with T‐DNA insertion in the first exon corresponding to the 5’ untranslated region of *NDR7*
*ndr8* (SALK_075540C)	Line with T‐DNA insertion in the eleventh intron of *NDR8*
*ndr47*	*NDR4 NDR7* double‐mutant obtained by the cross between *ndr4* and *ndr7* plants
*ndr68*	*NDR6 NDR8* double‐mutant obtained by the cross between *ndr6* and *ndr8* plants
*ndr467*	*NDR4 NDR6 NDR7* triple‐mutant obtained by the cross between *ndr47* and *ndr6* plants
*ndr478*	*NDR4 NDR7 NDR8* triple‐mutant obtained by the cross between *ndr47* and *ndr8* plants
*ndr46+−78*	Line that is homozygous for T‐DNA insertion in *NDR4*, *NDR7*, and *NDR8* and that is heterozygous for T‐DNA insertion in *NDR6*
*ndr4678+−*	Line that is homozygous for T‐DNA insertion in *NDR4*, *NDR6*, and *NDR7* and that is heterozygous for T‐DNA insertion in *NDR8*

### Genomic PCR

Genomic DNA solutions were prepared from cauline leaves of plants as previously described [21] and used as templates. PCR was run with these templates, the KOD FX Neo DNA polymerase, and the primers listed in Table S3.

### Accession numbers and a phylogenetic analysis

Details regarding the sequences of the genes used in this study are obtained with the following Arabidopsis Genome Initiative accession numbers: AT4G14350 (*NDR1*), AT1G03920 (*NDR2*), AT3G23310 (*NDR3*), AT2G19400 (*NDR4*), AT2G20470 (*NDR5*), AT4G33080 (*NDR6*), AT1G30640 (*NDR7*), AT5G09890 (*NDR8*), AT3G14370 (*WAG2*), AT4G04720 (*CPK21*), AT1G43700 (*VIP1*), and AT3G62250 (*UBQ5*). A phylogenetic tree was generated by ‘One click workflows’ for ‘Phylogeny Analysis’ on NGPhylogeny.fr (https://ngphylogeny.fr/) [22] with the amino acid sequences of NDR1‐8 and CPK21 as input.

## Results

### NDR2, NDR3, and NDR8 phosphorylate VIP1 *in vitro*

**Fig. 1:**
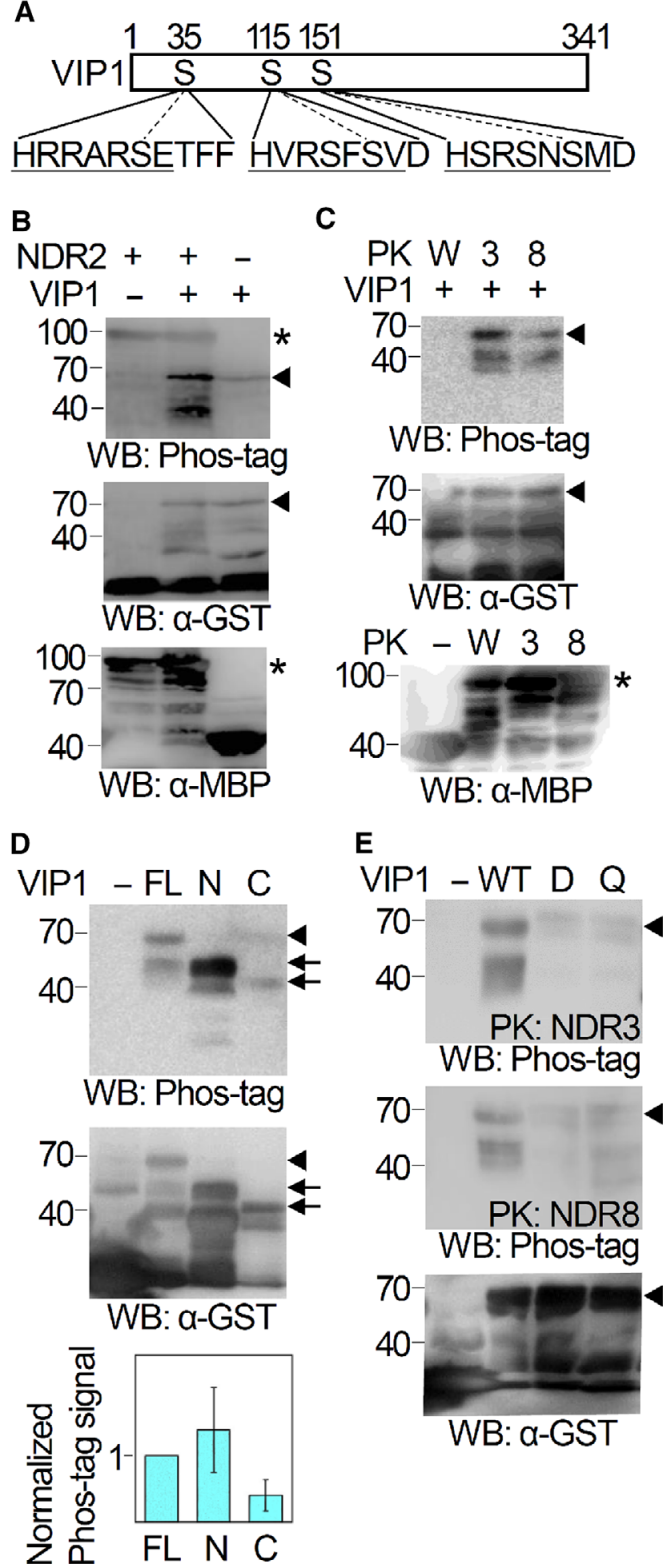
NDR2, NDR3, and NDR8 phosphorylate VIP1 *in vitro*. (A) VIP1 has putative NDR/LATS‐family PK phosphorylation sites. Amino acid positions in VIP1 are indicated at the top. S35, S115, S151, and their flanking sequences are presented at the bottom. The Hx[RK]xx[ST] consensus sequence for NDR/LATS‐family PK phosphorylation is underlined. (B–E) *In vitro* phosphorylation assays with MBP‐fused PKs and GST‐VIP1. The proteins were analyzed by western blotting with Phos‐tag biotin, an anti‐MBP antibody, or an anti‐GST antibody (‘WB: Phos‐tag’, ‘WB: α‐MBP’, or ‘WB: α‐GST’, respectively). Arrowheads indicate positions of GST‐VIP1. Asterisks indicate positions of MBP‐fused PKs. The GST‐fused proteins and MBP‐fused proteins were detected as ladders, and this may be due to unexpected degradation or incomplete translation of those proteins. The numbers on the left side of the images indicate molecular mass. The experiments were performed three times and a representative result is presented. (B) NDR2 phosphorylates VIP1 *in vitro*. MBP‐NDR2 and GST‐VIP1, which are indicated as ‘NDR2’ and ‘VIP1’, respectively, in the figure, were reacted and analyzed by western blotting. The presence and absence of these proteins in the reaction mixtures are indicated as ‘+’ and ‘−’, respectively. For ‘NDR2 −’, MBP alone was used instead of MBP‐NDR2. For ‘VIP1 −’, GST alone was used instead of GST‐VIP1. (C) NDR3 and NDR8 also phosphorylate VIP1 but WAG2 does not. MBP‐NDR3, MBP‐NDR8, or MBP‐WAG2, which is indicated as ‘3’, ‘8’ or ‘W’, respectively, in the figure, was reacted with GST‐VIP1 and analyzed by western blotting. The presence and absence of GST‐VIP1 in the reaction mixtures are indicated as ‘+’ and ‘−’, respectively. For ‘VIP1 ‐’, GST alone was used instead of GST‐VIP1. For ‘WB: Phos‐tag’ and ‘WB: α‐GST’ (top and middle images, respectively), signals of proteins on the same membrane are presented. MBP‐NDR3, MBP‐NDR8, and MBP‐WAG2 were detected on a different membrane, and their signals are presented separately in the bottom image (for ‘WB: α‐MBP’). (D) NDR8 phosphorylates VIP1N rather than VIP1C. GST‐VIP1, GST‐VIP1N, or GST‐VIP1C, which is indicated as ‘FL’, ‘N’, or ‘C’, respectively, in the figure, was reacted with MBP‐NDR8 and analyzed by western blotting. For ‘VIP1 −’, GST alone was used. Arrows indicate positions of GST‐VIP1N and GST‐VIP1C. For the bottom panel, levels of Phos‐tag biotin‐derived signals for GST‐VIP1N and GST‐VIP1C were normalized by GST‐VIP1 signals obtained on the membrane with Phos‐tag biotin and on the membrane with an anti‐GST antibody. Data are means ± SD from three replicates. (E) NDR3 and NDR8 phosphorylate S35 and S115 of VIP1. GST‐VIP1, GST‐VIP1D (GST‐fused VIP1 variant that has Ser → Ala substitutions at the amino acid positions 35 and 115 of VIP1), or GST‐VIP1Q (GST‐fused VIP1 variant that has Ser → Ala substitutions at the amino acid positions 35, 115, 149, and 151 of VIP1), which is indicated as ‘WT’, ‘D’, or ‘Q’, respectively, in the figure, was reacted with either MBP‐NDR3 (for the top panel, ‘PK: NDR3’) or MBP‐NDR8 (middle, ‘PK: NDR8’) and analyzed by western blotting. For ‘VIP1 −’, GST alone was used.

Serine residues at the amino acid positions 35, 115, and 151 (S35, S115, and S151, respectively) of VIP1 are its putative phosphorylation sites and a part of canonical 14‐3‐3 interaction motifs [17, 18]. We found that all of these residues are also a part of the Hx[RK]xx[ST] consensus motif (calculated probability is 0.0005) for the NDR/LATS PK‐dependent phosphorylation (Fig. 1A) and were tempted to determine whether VIP1 is in fact phosphorylated by NDR/LATS‐family PKs. NDR2, NDR3, and NDR8 are phylogenetically distant from each other [16] (see also Fig. S1) and may have different substrate specificity. These proteins were chosen for *in vitro* phosphorylation assays with VIP1. In western blotting with the phosphorylated protein probe Phos‐tag biotin, GST‐VIP1 signals could be detected when GST‐VIP1 was reacted with MBP‐NDR2, MBP‐NDR3, or MBP‐NDR8 *in vitro*, but not when GST‐VIP1 was reacted with MBP‐fused WAG2, an Arabidopsis PK that belongs to the AGC superfamily but that does not belong to the NDR/LATS family (Fig. 1B,C). GST‐VIP1N (1–186 amino acids of VIP1 fused with GST) was detected as strong signals by Phos‐tag biotin when reacted with MBP‐NDR8, but GST‐VIP1C (165–341 amino acids of VIP1) was detected as weaker signals (Fig. 1D). GST‐fused VIP1 S35 115A, in which VIP1 S35 and S115 are both substituted by alanine, caused weaker Phos‐tag biotin‐derived signals than GST‐VIP1 when reacted with either MBP‐NDR3 or MBP‐NDR8 (Fig. 1E, ‘VIP1 D’). GST‐fused VIP1 S35 115 149 151A, in which VIP1 S35, S115 S151, and the serine residue at position 149 (S149) are all substituted by alanine, also caused weaker Phos‐tag biotin‐derived signals as did VIP1 S35 115A (Fig. 1E, ‘VIP1 Q’). The banding patterns of ‘VIP1 D’ and ‘VIP1 Q’ were similar to each other. These results suggest that either or both VIP1 S35 and S115 is/are phosphorylated by the Arabidopsis NDR/LATS‐family PKs *in vitro* and that neither VIP1 S149 nor S151 is phosphorylated by those PKs at least *in vitro*.

### Expression patterns of *NDR1‐8*

**Fig. 2:**
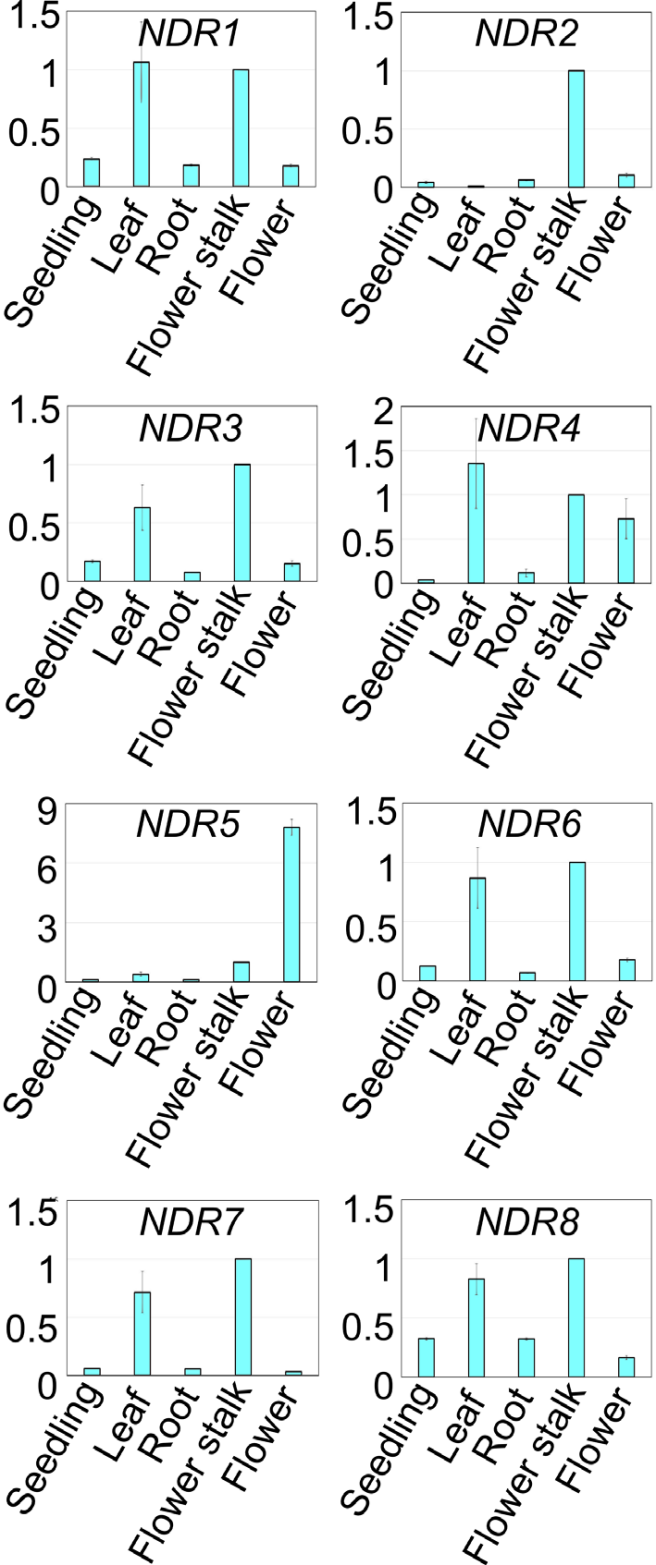
Expression patterns of *NDR1‐8*. Relative expression levels of *NDR1‐8* were analyzed by quantitative RT‐PCR using *UBQ5* as the internal control gene. Data are means ± SD from of three biological replicates.

In RT‐PCR, expression of *NDR1‐8* was detected in all the tissues studied (i.e., seedlings, rosette leaves, roots, flower stalks, and flowers). *NDR1*, *NDR3*, *NDR4*, *NDR6*, *NDR7*, and *NDR8* were expressed strongly in rosette leaves and flower stalks and weakly in the other tissues. *NDR2* was expressed strongly in flower stalks and weakly in the other tissues. *NDR5* was expressed strongly in flowers and weakly in the other tissues (Fig. 2).

### An *NDR4 NDR6 NDR7 NDR8* quadruple mutation causes embryonic lethality

**Fig. 3:**
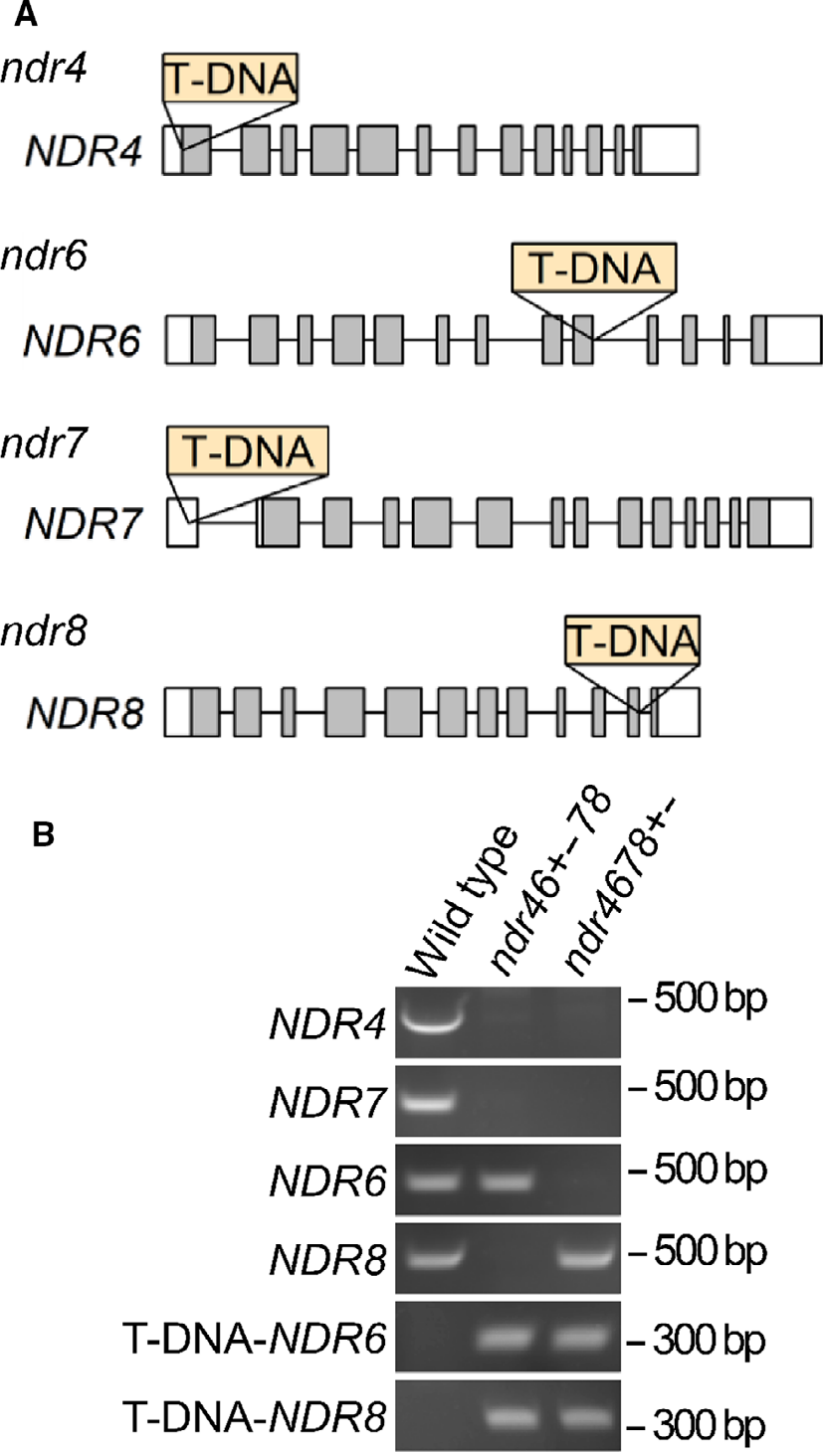
T‐DNA insertion lines for *NDR4*, *NDR6*, *NDR7*, and *NDR8*. (A) T‐DNA insertion positions in *ndr4*, *ndr6*, *ndr7*, and *ndr8*. Boxes indicate exons. White and gray regions in the boxes indicate untranslated regions and coding sequences, respectively. (B) Genomic PCR analyses of T‐DNA insertion in *ndr46+−78* and *ndr4678+−* plants. For ‘T‐DNA‐NDR6’, T‐DNA and its flanking sequence containing *NDR6* were analyzed. For ‘T‐DNA‐NDR8’, T‐DNA and its flanking sequence containing *NDR8* were analyzed.

NDR4, NDR6, and NDR8 are phylogenetically close to each other, and NDR7 is distant from the other NDR/LATS‐family PKs [16] (see also Fig. S1). This led us to hypothesize that *NDR4*, *NDR6*, and *NDR8* together play a specific physiological role and that *NDR7* also plays a specific physiological role. To test this idea, Arabidopsis mutant lines carrying T‐DNA insertions in these NDR/LATS PK genes were generated (Table 1; Fig. 3). No growth defect was found in any of these lines, including *ndr68*, *ndr478*, and *ndr467*, under a normal growth condition. However, although selfing *ndr46+−78* plants is expected to generate *ndr46−−78* (i.e., *NDR4 NDR6 NDR7 NDR8* quadruple mutant) plants and *ndr46+−78* and *ndr46++78* (i.e., *ndr478*) plants in the 1 : 2 : 1 ratio in the progeny, the numbers of these plants obtained were 0, 50, and 25, respectively. Similarly, selfing *ndr4678+−* plants is expected to generate *ndr4678−−* (i.e., NDR4 NDR6 NDR7 NDR8 quadruple mutant) and *ndr4678+−* and *ndr4678++* (i.e., *ndr467*) plants in the 1 : 2 : 1 ratio in the progeny, but the numbers of these plants obtained were 0, 48, and 24, respectively. These results suggest that an *NDR4 NDR6 NDR7 NDR8* quadruple mutation does not cause gametophytic lethality but that such a mutation causes embryonic lethality.

## Discussion

Our data indicate that at least NDR2, NDR3, and NDR8 can phosphorylate VIP1 *in vitro* but WAG2 cannot (Fig. 1B). This supports the idea that plant NDR/LATS‐family PKs differ in substrate specificity from the other plant AGC PKs. This idea is consistent with a previous finding that among members of the AGC superfamily, only NDR/LATS‐family members require H at the beginning of their phosphorylation sites [11]. On the other hand, even though CPK21 is not an AGC PK, it can also phosphorylate VIP1 *in vitro* [14]. Further studies are necessary to elucidate the substrate specificity of CPK21 and plant NDR/LATS‐family PKs.

Unlike the *NDR2 NDR4 NDR5* triple mutation [16], the *NDR4 NDR6 NDR7 NDR8* quadruple mutation was shown to cause embryogenic lethality. NDR4, NDR6, NDR7, and NDR8 are phylogenetically closer to each other than to the other NDR/LATS‐family PKs in Arabidopsis (Fig. S1). Because *NDR2*, *NDR4*, and *NDR5* are more strongly expressed in pollen grains than in other tissues [16] and because the other NDR/LATS‐family PK genes are not [16] (Fig. 2), the difference in physiological roles for the NDR/LATS‐family PK genes may be due to their expression patterns.

Although VIP1 can be a substrate of at least NDR2, NDR3, and NDR8 (Fig. 1), VIP1 is unlikely involved in the embryogenic lethality caused by the *NDR4 NDR6 NDR7 NDR8* quadruple mutation. This is because targeting of VIP1 to the nucleus, which could be promoted by the *NDR8* mutation, causes growth retardation but does not cause embryonic lethality [17, 23]. MOB1A and MOB1B both regulate not only pollen development but also vegetative growth [24] and can bind at least NDR2, NDR4, and NDR5 in the cytosol [16]. Thus, MOB1A and MOB1B may also play roles in the NDR/LATS‐family PK‐dependent regulation of embryogenesis.

Although NDR2, NDR3, and NDR8 can phosphorylate VIP1 *in vitro* (Fig. 1), previous proteome analyses detected NDR2 and NDR3 as well as NDR1 in both the plasma membrane and the cytosol, and NDR8 in the cytosol [25, 26]. Thus, substrates of NDR2 and NDR3 in cells may be different from substrates of NDR8. Further studies are necessary to elucidate substrates and physiological functions of plant NDR/LATS‐family PKs.

## Conflict of interest

The authors declare no conflict of interest.

## Author contributions

All authors conceived and designed the project. HSY, KF, SL, and DT acquired the data. HSY, TT, and DT analyzed and interpreted the data. HSY and DT wrote the paper.

## Supporting information

**Fig. S1**. A rooted phylogenetic tree for the NDR/LATS‐family PKs (i.e., NDR1‐8) in Arabidopsis.CPK21 was used as an outgroup. Amino acid sequences of NDR1‐8 and CPK21were aligned and built into the phylogenetic tree by“Oneclick workflows”for“Phylogeny Analysis”onNGPhylogeny.fr (https://ngphylogeny.fr/) [22].
**Table S1**. Primers used to clone *NDR2*, *NDR3*, *NDR8* and *WAG2* into pMAL‐c5E.
**Table S2**. Primers used for quantitative RT‐PCR.
**Table S3**. Primers used for genomic PCR.

## Data Availability

The data that support the findings of this study are available in Figs 1, 2, 3 and the supplementary material of this article.
