# Supplementary material for: NDR/LATS‐family protein kinase genes are indispensable for embryogenesis in Arabidopsis
Source: FEBS Open Bio. 2021 Aug 6;11(9):2600–6. doi: 10.1002/2211-5463.13257 (PMC8409290; doi:10.1002/2211-5463.13257)
Supplement: Supplementary file 1 — Fig. S1. A rooted phylogenetic tree for the NDR/LATS‐family PKs (i.e., NDR1‐8) in Arabidopsis.CPK21 was used as an outgroup. Amino acid sequences of NDR1‐8 and CPK21were aligned and built into the phylogenetic tree by“Oneclick workflows”for“Phylogeny Analysis”onNGPhylogeny.fr (https://ngphylogeny.fr/) [22]. Table S1. Primers used to clone NDR2, NDR3, NDR8 and WAG2 into pMAL‐c5E. Table S2. Primers used for quantitative RT‐PCR. Table S3. Primers used for genomic PCR. [file FEB4-11-2600-s001.pdf]

Supplementary materials for:

NDR/LATS-family protein kinase genes are indispensable for embryogenesis in Arabidopsis

Hyuk Sung Yoon[1], Kaien Fujino[2], Shenkui Liu[3], Tetsuo Takano[1], and Daisuke Tsugama[1*]

[1]Asian Research Center for Bioresource and Environmental Sciences (ARC-BRES), Graduate School of Agricultural and Life Sciences, The University of Tokyo, 1-1-1 Midori-cho, Nishitokyo-shi, Tokyo 188-0002, Japan

[2]Laboratory of Crop Physiology, Research Faculty of Agriculture, Hokkaido University Kita 9 Nishi 9 Kita-ku, Sapporo-shi, Hokkaido 060-8589, Japan

[3]State Key Laboratory of Subtropical Silviculture, Zhejiang A & F University, Lin'an, Hangzhou 311300, P.R. China

Table S1. Primers used to clone *NDR2*, *NDR3*, *NDR8* and *WAG2* into pMAL-c5E.

Primer name	Sequence (5' > 3')*	Restriction sites used in pMAL-c5E
NDR2_CDS_NotI_F	CTGTGCGGCCGCATGGATTCTGCAAGAAGTTGGTTTC	*Not*I
NDR2_CDS_NotI_R	CACCGCGGCCGCGGATCCTACTCAGAATCTAGACGTCTCAG	
NDR3_CDS_NotI_F	CTGTGCGGCCGCATGGATACTGCCAGAGCATGGC	*Not*I
NDR3_CDS_NotI_R	AGGAGCGGCCGCGGATCCTTAGGATGTGGCATTGTCGAAACG	
NDR8_CDS_NotI_F	AAGAGCGGCCGCATGGACGGCGCCGATGGAACCGTTCG	*Not*I
NDR8_CDS_NotI_R	CCCTGCGGCCGCGGATCCTATGTCTTGTGGTTTAACTCACC	
WAG2_CDS_NcoI_F	CCCTCTAGAGCCATGGAACAAGAAGATTTCTATTTC	*Nco*I and *Sal*I
WAG2_CDS_SalI_R	CCCGTCGACCAACGCGTTTGCGACTCGCGTAGC	

*Restriction sites are underlined

Table S2. Primers used for quantitative RT-PCR.

Primer name	Sequence (5' > 3')	Target
NDR1_RT_F	TCACCCTTGGTTTAGAGGCAC	*NDR1*
NDR1_RT_R	ATGGACCTGACTTGGCTGAC	
NDR2_RT_F	AAGTCCCCGGTATAGCCGAA	*NDR2*
NDR2_RT_R	CACGAGGCGTCGGATTAGAA	
NDR3_RT_F	GCCAAAGCGGCCATCAATTA	*NDR3*
NDR3_RT_R	CGAAACGTGTGGTGGAAGATG	
NDR4_RT_F	CGATGAGGTGGAGTGTCCAA	*NDR4*
NDR4_RT_R	GTGGAGGTGAAACGCTTCCT	
NDR5_RT_F	CCTGGAATGGCGGAGTTGAA	*NDR5*
NDR5_RT_R	TTCCCTGAACAACCGGAGGA	
NDR6_RT_F	GGTTCAAGGATGTTGTGTGGG	*NDR6*
NDR6_RT_R	GCATCTTCCTGGAGAGTCCTG	
NDR7_RT_F	CGGAACTGAAGCGGAAGAGTA	*NDR7*
NDR7_RT_R	TGGTGATCCGGAAGACCCTC	
NDR8_RT_F	GCATCCATGGTTCAAAGGCA	*NDR8*
NDR8_RT_R	GGACCAACTTGTGGTGCTTC	

Table S3. Primers used for genomic PCR.

Primer name	Sequence (5' > 3')	Target*
NDR4_G_F	TGACCAAGGAAAGAGAAATGCG	*NDR4*
NDR4_G_R	CCCAACGCCTGCAAATCAAA	*NDR4*
NDR6_G_F	TGCAACGTTGACCATAGGCT	*NDR6*
NDR6_G_R	TCACCATCATCACCAGTGGC	*NDR6* and T-DNA-*NDR6*
NDR7_G_F	TCCGTTCAATCGTGCTGTCA	*NDR7*
NDR7_G_R	ATACGCACAAACACCATGCG	*NDR7*
NDR8_G_F	GACGTCCAAGGACACCAACT	*NDR8* and T-DNA-*NDR8*
NDR8_G_R	CTCTCCATTGCCTCTGCCAA	*NDR8*
T-DNA_LBb1.3	ATTTTGCCGATTTCGGAAC	T-DNA-*NDR6* and T-DNA-*NDR8*

*Target names are consistent with Fig. 1B.

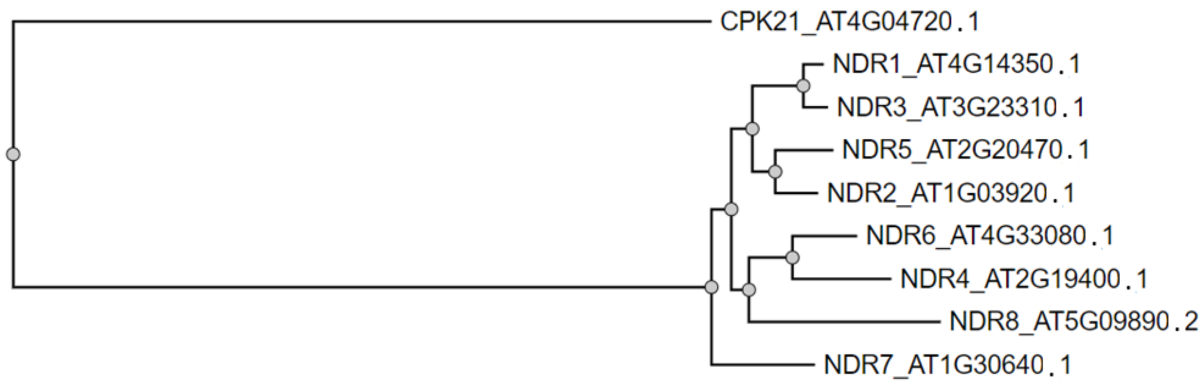

Figure S1. A rooted phylogenetic tree for the NDR/LATS-family PKs (i.e., NDR1-8) in Arabidopsis. CPK21 was used as an outgroup. Amino acid sequences of NDR1-8 and CPK21 were aligned and built into the phylogenetic tree by “One click workflows” for “Phylogeny Analysis” on NGPhylogeny.fr (https://ngphylogeny.fr/) [22].
